# Obstetric violence: perspectives from mothers, midwives, and obstetricians

**DOI:** 10.3389/fgwh.2025.1609632

**Published:** 2025-06-05

**Authors:** Zaira Reyes-Amargant, Concepció Fuentes-Pumarola, Marta Roqueta-Vall-llosera, Josep Garre-Olmo, David Ballester-Ferrando, Carolina Rascón-Hernán

**Affiliations:** ^1^ Health, Gender and Aging Research Group, Department of Nursing, University of Girona, Girona, Spain; ^2^ Catalan Health Institute, Girona, Spain; ^3^ Research Group on Development of the Nursing Profession, Department of Nursing, University of Girona, Girona, Spain

**Keywords:** obstetric violence, maternal health, pregnancy, patient-provider communication, informed consent, health care quality, midwifery, qualitative research

## Abstract

**Background:**

According to the World Health Organization (WHO), the majority of the 140 million annual births occur without complications. Women desire a positive birth experience based on respectful care, clear information, and emotional support, which enables them to make informed decisions and maintain control over their reproductive process. However, many women experience disrespectful or abusive treatment during obstetric care, with lasting consequences for both their physical and mental health. This study explores the factors that influence respectful maternal care and the phenomenon of obstetric violence, as perceived by mothers, midwives, and obstetricians.

**Methodology:**

A qualitative study using a phenomenological approach was conducted in eight public and private hospitals in the Girona Health Region (Catalonia, Spain) between 2021 and 2022. In-depth interviews were conducted with eight mothers and eight healthcare professionals (midwives and obstetricians) selected through purposive sampling. The interviews were transcribed verbatim and analysed using thematic analysis to identify meaningful units and key themes.

**Results:**

The narratives collected allowed for the identification of key elements of non-respectful maternal care. A lack of information during pregnancy and childbirth emerged as a central concern for both mothers and professionals, affecting their sense of control and satisfaction. Poor communication with healthcare providers, particularly with anaesthesiologists and obstetricians, was perceived as a barrier to quality care. Mothers reported experiences of disrespect, the absence of informed consent for procedures such as episiotomies, and paternalistic treatment. Obstetricians showed resistance to the term “obstetric violence,” while midwives recognized it as practices and attitudes that negatively affect the maternal experience.

**Conclusions:**

The discrepancy between professional perspectives and mothers' experiences highlights the urgent need for transformation in obstetric care. It is crucial to promote a care model based on evidence, effective communication, and respect for women's rights. Incorporating a gender perspective into healthcare training and strengthening public policies to ensure respectful obstetric care are essential measures to improve the quality of care and prevent obstetric violence.

## 1 Introduction

According to the World Health Organization (WHO), approximately 140 million births occur worldwide each year, many of which take place without risk factors or complications for the mothers and their babies (1). Women desire a positive childbirth experience, which refers to interactions and events directly related to childbirth that provide continuous care and emotional support, exceed their personal expectations, ensure a healthy and psychologically safe environment, and allow them to maintain a sense of control and autonomy in making informed decisions even, when medical interventions are required. Additionally, women want to feel respected by healthcare professionals and trust their technical competence (1–3). To enhance the quality of childbirth experiences, it is essential to adopt a woman-centered care approach that integrates a holistic perspective rooted in human rights principles (1, 4)*.*

Unfortunately, many women experience disrespectful and abusive treatment during childbirth in facilities worldwide. Such treatment not only violates the rights of women to respectful care but can also threaten their rights to life, health, bodily integrity, and freedom from discrimination. The lack of standardized definitions, instruments, and research methodologies in studies assessing mistreatment in maternity care has likely led to systematic inaccuracies in reported prevalence rates. As a result, prevalence estimates vary widely, ranging from 15.2% in India (5) to 78.4% in Italy (6). In Spain, 67.4% of women reported experiencing obstetric violence, with physical violence being the most frequently reported form (54.5%) (7). By contrast, another study found that 38% of women perceived themselves as having experienced obstetric violence; however, the authors suggest this figure may be underestimated due to underreporting or lack of awareness among participants (8). While improvements in the quality of care have led to an increase in medical interventions, this has also been recognized as a potential barrier to patient satisfaction (9).

The literature contains many terms to refer to this disrespect towards women during pregnancy, childbirth, and postpartum processes; obstetric violence, institutional violence, disrespectful or offensive treatment, medical authoritarianism, dehumanized treatment, abuse of medicalization and pathologization of physiological processes, lack of respect and abuse, mistreatment of women during childbirth, among others (8, 10–20). Although the concepts are morphologically different, they share the fact of being a form of gender-based violence, stemming from gender inequalities and compromising women's human rights (21).

Disrespect, mistreatment and obstetric violence significantly impact on women, and have been associated with birth trauma, postpartum depression, post-traumatic stress disorder, negative implications on sexuality, exacerbated risks of complications during childbirth, and distrust in the health system, resulting in unwillingness to seek medical care (1, 4, 11, 14, 19, 20, 22).

For these reasons, this study aims to explore the factors that influence respectful maternal care and the phenomenon of obstetric violence, as perceived by mothers, midwives, and obstetricians. With these results, we aim to enhance the existing body of knowledge, given the scarcity of studies in our context that explore respectful maternal care. In this way, the information gathered may contribute to the improvement of clinical guidelines for maternal care.

## 2 Method

### 2.1 Study design and setting

This study follows a qualitative design with a phenomenological approach, aiming to explore and understand the lived experiences of mothers and healthcare professionals during childbirth. Phenomenology seeks to capture how individuals make sense of their experiences, emphasizing subjective interpretations (23, 24). The study was conducted between 2021 and 2022 in all eight public and private hospitals of the Health Region in the province of Girona (Northeast Catalonia, Spain), which provide healthcare services to 870,483 inhabitants across 218 municipalities (25). In Spain, all women have access to free public maternal care. Most births occur in public hospitals (81.4%), while 18.6% take place in private facilities. Home births are rare (1.6%) and are not covered by the public healthcare system (26). In public hospitals, midwives primarily manage low-risk births, with obstetricians intervening in cases of complications or when surgical interventions are necessary. Conversely, in private hospitals, obstetricians typically lead childbirth care (27). Due to a national shortage of midwives—currently 6.1 per 10,000 women—achieving a 1:1 midwife-to-woman ratio during labour is unfeasible (28). Our study includes births attended in both public and private hospital settings, where midwives and obstetricians share delivery spaces.

### 2.2 Sample

Eight mothers and eight professionals were selected through purposive sampling, ensuring diversity in representation across different childbirth care centers in the Health Region. To guarantee the diversity of professionals, factors such as years of experience, type of institution where they work (public or private), and gender were considered. In the case of mothers, to capture a variety of profiles, variables such as age, origin, educational background, type of institution where they gave birth, parity, and type of birth were considered. The inclusion criteria for women were being over 18 years old. The exclusion criterion was a lack of proficiency in Spanish. For healthcare professionals, the inclusion criteria were willingness to participate in the study and signing informed consent. Tables 1, 2 show the sociodemographic characteristics of the participants.

**Table 1:** Sociodemographic characteristics of the mother's participants.

Code	Age	Origin	Education	Facility	Parity	Birth
M1	28	Center/South Africa	Primary	Public	Multiparous	Emergent caesarean
M2	33	Spain	University	Private	Nulliparous	Vaginal
M3	20	Brazilian	Secondary	Public	Nulliparous	Vaginal
M4	40	Spain	University	Public	Nulliparous	Caesarean
M5	29	Spain	Primary	Public	Multiparous	Vaginal
M6	31	Spain	University	Public	Nulliparous	Caesarean
M7	31	Spain	Secondary	Public	Multiparous	Urgent Caesarean
M8	42	Spain	University	Public	Nulliparous	Instrumental

**Table 2:** Sociodemographic characteristics of the professional's participants.

Code	Experience (years)	Center	Gender
O1	20	Public	Man
O2	23	Public and Private	Man
O3	9	Public	Woman
O4	6	Public and Private	Woman
Mi1	11	Public	Woman
Mi2	27	Public	Woman
Mi3	4	Public	Woman
Mi4	39	Private	Woman

Code: O, obstetricians; Mi, midwives.

### 2.3 Procedure

Mothers: Each candidate was invited to participate in the study by a midwife during her hospitalization, usually one day after birth. After signing the informed consent form, one of the researchers contacted the participants by phone to arrange a date and time for the interview. One interview was conducted face-to-face, while the remaining seven took place via Microsoft Teams. All interviews lasted approximately 40–60 min and were conducted within the first six months after childbirth.

Professionals: The obstetrics departments of each hospital in the Girona region were contacted, and the participation of one obstetrician or midwife per centre was requested. After signing the informed consent form, a date and time for the interview were arranged. Three interviews were conducted face-to-face, and five were held via Microsoft Teams. The duration of each interview was between 40 and 60 min.

The interviews were conducted by four researchers (one man and three women), three of whom are experts in qualitative research (PhD), and one a PhD student. One of the researchers, a midwife, did not participate as an interviewer or observer to avoid potential bias. Before starting the interviews, the script questions and interview procedures were discussed and reviewed. Each interview was conducted by two researchers: an interviewer and an observer. The observer played a passive role, refraining from participating in the discussion. Instead, they focused on non-verbal aspects and the interview context, aiding in the later analysis of participants' narratives and providing a more comprehensive perspective (24).

The interviews were conducted in Spanish, recorded, and subsequently translated into English. Each translation was reviewed by a professional translator. Data collection continued until theoretical saturation was achieved.

### 2.4 Instrument

Data were collected through in-depth interviews using a semi-structured guide, which allowed for the exploration of key topics while maintaining flexibility to follow the participants' emerging narratives. Table 3 show the women's interview and Table 4 show the midwives and obstetricians' interview.

**Table 3:** The women's’ interview.

Questions
- Are you satisfied overall with the care you received from all the professionals (midwives, obstetricians, administrative staff..) during your pregnancy, delivery and the postpartum period?
- Did the professionals guide you in creating your birth plan? Do you think your birth plan was respected? Do you think you were the protagonist of your labour and delivery? Do you believe you participated in the decision-making process during labour?
- Did you feel that you participated in the decision-making process after the baby was born? For example, were you able to do skin-to-skin contact, decide when you wanted the umbilical cord to be cut, etc.?
- Do you consider that they always asked for your consent and informed you before performing any procedure during childbirth?
- Do you consider that you received the necessary information about pregnancy, labour and postpartum period?
- If an episiotomy (perineal cut) was performed during childbirth, were you asked for consent before carrying it out? Were you informed about why it was necessary, and did you agree to it? How did you experience it?
- Do you consider that there was good communication between you, the person accompanying you, and the healthcare staff?
- Did they ever use words that you didn't understand and left you with doubts during pregnancy, delivery and the postpartum period? At any moment, were disqualifying or ironic tones used regarding your person or your behaviour?
- Do you consider that you received good treatment during labour from the professionals who attended you? If not, do you believe this is due to any specific aspect?
- Were any procedures performed on the new born without your or your partner's presence (such as omissions, heel prick test, etc.)? If so, what justifications did the healthcare staff provide? And how did you experience it? Were any options given to you?
- Have you felt violated by the healthcare professionals at any point during pregnancy, labour or the postpartum period?
- Regarding your pregnancy and childbirth.. Do you think you received individualized care based on your personal needs or characteristics (culture, beliefs, social situation, etc.)?
- Did you feel a lack of privacy at any point? If so, please explain what happened and how you felt.
- Did you feel judged or criticized by any member of the healthcare staff at any point as a result of the decisions made during the process?
- Do you think that, overall, you received respectful maternal care during pregnancy, birth and postpartum?

**Table 4:** The midwives and obstetricians’ interview.

Questions
- Do women have expectations and a birth plan? Do they share them with you? How do they communicate their plans, and what challenges do they face?
- Do you think woman's decisions are respected throughout the entire childbirth process?
- Regarding informed consent, are there situations where it is not requested? or do you always request it? When should it not be required?
- If a woman refuses a procedure that you believe it is necessary, how do you handle the situation?
- Do you think professionals provide enough information?
- Do you believe that women come to consultations already informed?
- Do you think there is good communication between the woman and the healthcare staff? Do you encounter any barrier regarding communication?
- Do you use technical jargon?
- Can women express themselves freely during the process of labour, pregnancy, and postpartum? Crying, shouting, communicating uncertainties..
- Do you think that in your facility all the practices carried out are based on the latest scientific evidence? Have you identified any practices used with low levels of evidence?
- Have you ever felt compelled to perform a procedure or pressured by another healthcare professional?
- What does obstetric violence mean to you?
- Have you ever felt that you caused pain or suffering to a woman unintentionally?
- Have you witnessed obstetric violence in your workplace?
- Do you believe that, at any point, you may have committed obstetric violence, even if unintentionally?

The interview scripts were conducted by a research team of six members (four women and two men) with backgrounds in nursing, midwifery, psychology, and anthropology, under the guidance of two psychologists specializing in gender and gender-based violence. A thorough literature review was carried out to identify key areas for exploration regarding maternity care. These areas were discussed by the team in multiple rounds until a consensus was reached.

### 2.5 Data analysis

A thematic analysis was introduced by Braun and Clarke (29) approach. This process involved the literal transcription of the interviews, repeated readings of the transcripts to familiarize researchers with the data, and the identification of meaning units. These units were then grouped into themes and categories that reflect the participants' essential experiences. Data coding was carried out independently by three researchers, and the results were compared. Any discrepancies were discussed until a consensus was reached, enhancing the reliability of the coding process.

For the analysis of the discourse, each interviewee was assigned a letter and a number: (M) for mothers, (Mi) for midwives, and (O) for obstetricians.

### 2.6 Ethical considerations

Participation in the interviews was voluntary. Participants signed a written informed consent form, ensuring respect for the principle of autonomy. They were informed about the study's purpose and how their data would be handled. The recordings were transcribed and stored on secure servers at the University of Girona. Once transcription was completed, the recordings were deleted. The study protocol was approved by the Ethics Committee of Research with Medicines (CEIm Girona, reference 2021.043).

All data were treated with absolute confidentiality, and informed consent was obtained in accordance with the provisions of Regulation (EU) 2016/679 of the European Parliament and of the Council of April 27 on Data Protection (GDPR) (30), as well as Organic Law 3/2018 on Data Protection and Guarantee of Digital Rights (Article 6.1.a + 9.2.a GDPR, additional provision 17.2a LOPD-GDD) (31).

This study does not involve any high-risk data processing situations: it does not include data profiling or automated decision-making, the use of artificial intelligence tools, data exploitation techniques with Big Data technologies, biometric systems, or geolocation systems.

### 2.7 Rigor

This study was conducted following the COREQ checklist for study design, data collection, analysis, and publication (24) (Supplementary Material). A pilot interview was conducted beforehand to ensure the proper functioning of the interview process.

## 3 Results

This section presents the analysis of the discourses of women, midwives, and obstetricians. Three key themes emerged regarding their experiences and perceptions of maternal care: information, communication, and decision-making. Regarding the phenomenon of obstetric violence, we have gathered insights from healthcare professionals (midwives and obstetricians) alongside the experiences of mothers. The findings reveal that many women endure situations that may be classified as obstetric violence.

### 3.1 Experiences and perceptions of maternal care

**Figure 1:**
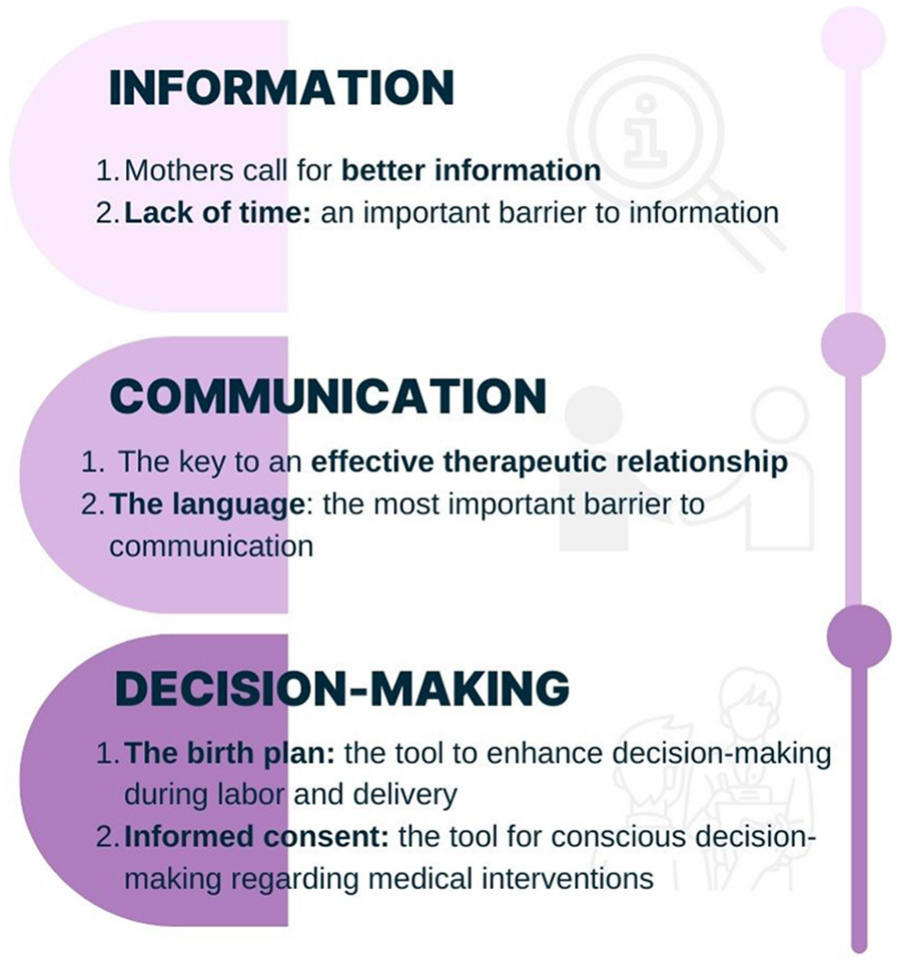
Themes and subthemes representing experiences and perceptions of maternal care.

Figure 1 shows themes and subthemes representing experiences and perceptions of maternal care**.**

#### 3.1.1 The information

##### 3.1.1.1 Mothers call for better information

According to professionals' opinions, women are increasingly requesting more information about childbirth processes. This trend may create expectations about motherhood that can lead to frustration, potentially affecting their satisfaction with and perception of maternal care.

“Over these years the patients have changed a lot. Women have gone from having no information to being not properly informed. And this often causes unrealistic expectations of childbirth that can lead to frustration” (Obstetrician2_O2_).

Access to information is perceived by mothers as a positive aspect of their childbirth experience. When mothers do not receive enough information from healthcare professionals, they often seek it from unreliable sources.

“As positive memories, I remember a nurse who was like a mother, you know? She explained everything to me, calming me down. I felt very supported” (Mother6_M6_).

“Yes, most of them misinform themselves. Through forums or Instagram, there's a lot of information but it's not always true” (Midwive3_Mi3_).

##### 3.1.1.2 Lack of time: an important barrier to information

Professionals and mothers agree that limited time during clinics is a barrier to providing accurate information.

“We strive to provide information, but it's important to remember that in an obstetric consultation, there is often limited time to fully explain everything” (Mi3).

“I missed a more relaxed approach. With the gyneacologist, I might go in with three questions, but I would only ask one because I always felt she was in a hurry. With the midwife, the visits were more relaxed; we talked more, and she listened more” (M1).

#### 3.1.2 The communication

##### 3.1.2.1 The key to an effective therapeutic relationship

Professionals agree that effective communication enhances patients' confidence in the procedures being performed, leading to a stronger therapeutic relationship between healthcare providers and mothers.

“When communication between the doctor and patient is strong, and the doctor clearly explains the reasons for proposing a medical intervention, the patient is more likely to understand and feel confident in the procedure” (O2).

In the public health system, mothers are not assigned a specific midwife or obstetrician as their primary caregiver. Instead, they interact with multiple professionals during labour and birth, a time when they are particularly vulnerable. As a result, there is insufficient time to establish a strong, trust-based relationship.

“Sometimes, the patient has only just met you in the birth room because labour isn't progressing as expected. In that moment, a decision must be made that she may not have wanted, which makes the situation more complicated” (O2).

“I suppose this is common throughout the public health system — not having a designated gynaecologist that women can always see. This creates a sense of unease for them” (O3).

Mothers and professionals report that communication needs improvement due to the poor communication skills of some midwives and obstetricians.

“I acknowledge that we can do better. I believe we must significantly improve our communication with them, as it is the key to everything” (O2).”

“I was already stressed, and the midwife only made it worse. She would tell my mother, “Tell her she needs to push harder,” and I felt frustrated. I just wanted to say, “This is my moment—don't talk to my mother, talk to me” (M3).

##### 3.1.2.2 The language: the most important barrier to communication

Some professionals also mention the challenges of communication when there is a language barrier.

“Of course, we try to explain, but it can be really exhausting when you explain something three times, only for them to ask the same question again because they still don't understand. Eventually, it reaches a point where it tests our patience as well” (Mi1).”

#### 3.1.3 The decision-making

##### 3.1.3.1 The birth plan: the tool to enhance decision-making during labour and birth

Some women report being unaware of the birth plan, while those who created one say it was not taken into consideration.

“I didn't do the birth plan, in fact, I was never informed about it” (M6).

“You go to the midwife, and she tells you that you have to make a birth plan. I filled it out but nobody at the hospital asked me for it. Everything happens on the go” (M4).

Professionals believe that birth plans can lead to an idealized view of childbirth or to demands and expectations that cannot always be met in the birth room.

“They are told that they can't come in with a fixed idea of how the birth will go because it can be totally different, that's how childbirth is. They have quite high expectations to translate this into the birth plan. These plans are becoming increasingly specific, and it's difficult to deal with this because there are things we can't provide at the hospital. For example, not inserting an intravenous line or not allowing anyone into the examination room” (O4).

“They have idealized expectations of childbirth, envisioning experiences like labouring in the shower, avoiding an epidural, and having a completely natural birth” (Mi2).

Mothers reported having limited decision-making power, as they are subject to healthcare protocols, which leads to a lack of control.

“You’re reliant on the doctor's judgment and the established protocol since they have the expertise. However, when things go wrong… I may not fully understand the protocol, but I believe it should be open to review” (M8).

Professionals affirm that women have the opportunity to make informed decisions, especially during pregnancy. However, during labour, if medical complications arise or disagreements occur, healthcare professionals may need to intervene.

“During pregnancy, if they don't want to get a vaccine or take vitamins, we explain what we recommend and if they don't want to, it's recorded, and obviously it's respected. There aren't many decisions to make during pregnancy, so we try to respect them, but if there's something that we medically consider it cannot be fulfilled, we tell them. For example, in childbirth, there are more and more people who don't want to have an intravenous line inserted, and now we have developed a protocol for refusing the insertion of the IV line” (O4).

##### 3.1.3.2 Informed consent: the tool for conscious decision-making regarding medical interventions

Written informed consent is mainly obtained for epidural anaesthesia and caesarean sections. For other procedures, consent is generally obtained verbally.

“We usually do get written informed consent for a caesarean section. In the case of an episiotomy, we just obtain a verbal one. And for epidural anaesthesia, yes, it is also signed” (O2).

Professionals report that informed consent would be needed for procedures such as amniotomies or instrumental deliveries.

“What I miss is the informed consent for an amniotomy. Which for me is a trivial thing, but for the woman it's not, and she needs to have all the information, the pros and cons” (Mi1).

Additionally, since signatures are obtained during vulnerable and painful moments, many women are unaware of what they are signing.

“The anaesthesiologist does make you sign the paper that you can't look at anything because you're there in an awkward position and with pain, and you're just wishing for them to give you the epidural, so you sign whatever they put in front of you” (M4).

### 3.2 The phenomenon of obstetric violence

#### 3.2.1 The midwives and obstetricians' point of view

**Figure 2:**
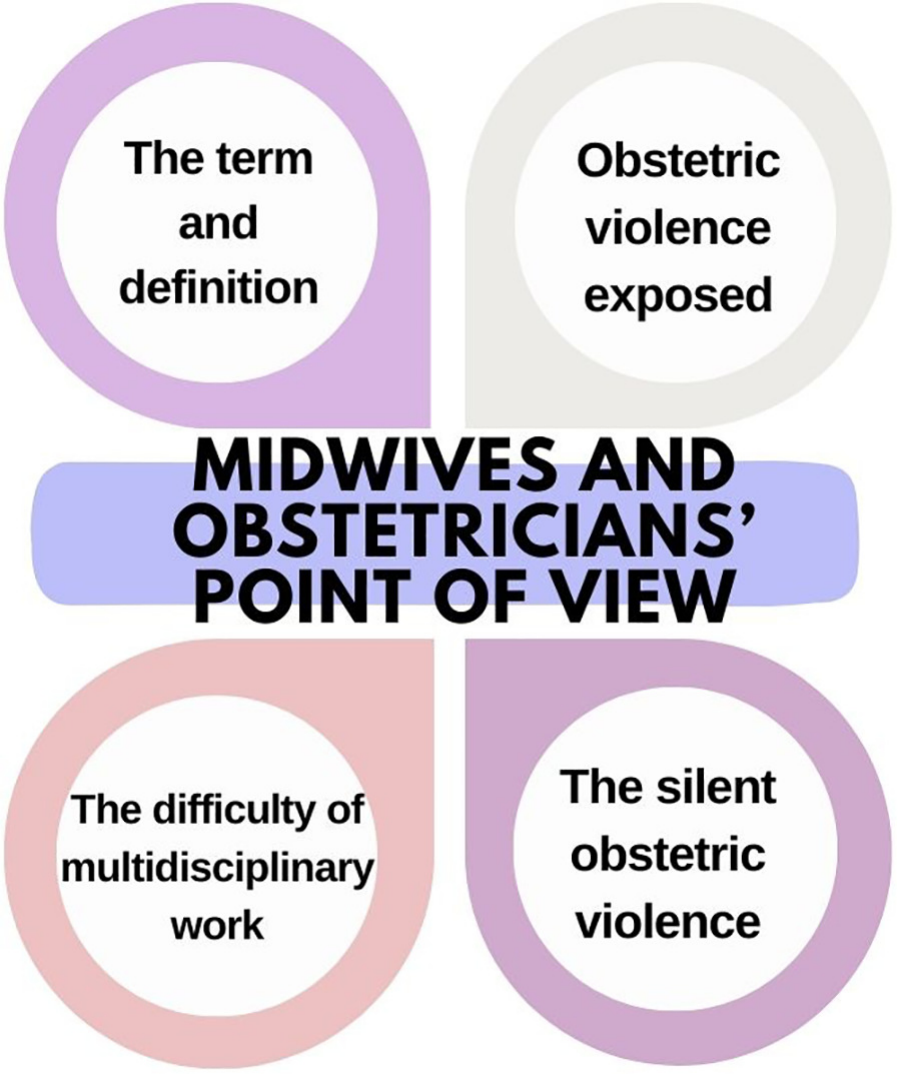
Themes representing midwives and obstetrician's point of view of obstetric violence.

Figure 2 shows themes representing midwives and obstetrician's point of view of obstetric violence.

##### 3.2.1.1 The term and the definition

Regarding professionals' opinions on obstetric violence, some emphasize that they do not like the term because they find it offensive.

“For us, the term obstetric violence it really annoys us because the word violence is a very hard word, which predisposes that someone is consciously doing something harmful to another person. We have no intention of causing pain or harm to a patient. On the contrary, our job is precisely to help mothers. So, this term obstetric violence, which is very trendy, we don't like it. We must accept that there are things we are not doing entirely right, that we need to improve them, of course” (O2).

Regarding the definitions, opinions vary. However, there is a consensus that when a medical procedure is performed against the mother's wishes, it is done because healthcare professionals deem it medically necessary, and therefore, it is not considered obstetric violence.

“Obstetric violence includes actions, techniques, and even verbal expressions that can emotionally impact a woman and affect her sensitivity. While the definition of violence can be subjective, it also encompasses subtle elements like words or comments. It's a term open to various interpretations.” (Mi2).

“ Obstetric violence involves performing unnecessary procedures, not merely acting against a mother's preferences. For example, while some mothers may not want an episiotomy, there are cases where it is medically necessary. In my view, that does not constitute violence” (Mi4).

“There is a fine line between medically necessary interventions and obstetric violence, and I believe many people struggle to distinguish between the two. Performing an episiotomy to prevent a severe perineal tear, for example, does not automatically constitute obstetric violence” (O4).

##### 3.2.1.2 Obstetric violence exposed

Although several professionals acknowledge that obstetric violence has occurred in birth rooms, or even that they have been responsible for it at some point, they state that it is becoming less frequent.

“I believe that obstetric violence was more prevalent in the past, but it has gradually declined over time” (O4).

“When I started, we performed the Kristeller's manoeuver and conducted vaginal examinations—often without asking—because it was considered normal practice at the time. Now, things have changed; we strive to do everything with respect” (Mi4).

“Those of us who are of a certain age see it as a professional flaw. Obstetric violence was not only permitted but was the standard practice. The patient's decisions carried very little weight; you had established criteria for what you had to do, and that was it. So, of course, we've had to gradually change our mindset. I think we're on an acceptably good path, but we still have a long way to go” (O2).

Some professionals assert that acts which could be considered obstetric violence may result from a lack of knowledge among healthcare providers or the low level of evidence that is sometimes applied in obstetrics.

“(Acts that are performed routinely) and that are likewise considered obstetric violence and that they don't know that the woman could avoid these situations.. I don't deny that these situations exist, the only thing that bothers me is that it's labelled as violence, because it seems as if the professional wants to do it on purpose, and many times it's due to lack of knowledge, isn't it?” (O3)”.

“Obstetrics is always based on very soft evidence. Perhaps, if we're being very precise, surely there are quite a few procedures that we do that are not entirely supported by evidence” (O1).

Professionals who have witnessed acts that could be considered obstetric violence:

“For example, the woman is pushing, everything is going well, and then the doctor arrives in a hurry. We do a kiwi (manual vacuum) and that's it, or an episiotomy, for example. You could have waited a bit, we've been here for an hour with the woman pushing, and it wouldn't have been more than 10 min. The baby was fine, but well, of course.” (Mi4).

“We have seen deliveries being instrumented when it wasn't necessary, just to teach a gynaecology resident.” (Mi1).

When directly asked if they have committed obstetric violence, professionals acknowledge instances where they have failed to alleviate pain or where there were an excessive number of professionals present when it was not necessary:

“When performing the suture and the anaesthesia is not effective, you tell her, “Try to hold on a bit, it's just three stitches” but it hurts her. If you break the waters, the contractions will be more painful. I've done it before, and I know it will hurt her” (Mi1).

“During the expulsive phase of labour, well, not always, but at specific moments when the gynaecologist, midwife, nursing assistant, and gynaecology resident students are present in the room.. Not in all births, but when it happens, I notice that there are too many people” (O3).

Additionally, they also refer to Kristeller's manoeuver:

“I committed obstetric violence in an extreme situation where the baby was at risk of being stillborn and the woman refused a caesarean section. There was a language and cultural barrier, and she wanted to give birth vaginally. We performed a Kristeller's manoeuver. Was it obstetric violence? Yes. Was it justified? Yes. Would I do it again? I think so” (Mi3).

“Kristeller's it's a technique that we don't like, and we try not to do it at all, but sometimes you see it as necessary for the woman and the child” (Mi4).

##### 3.2.1.3 The silent obstetric violence

Some professionals report that consent is not always required.

“With episiotomies, the procedure is often performed first and explained afterward. Consent isn't typically sought, likely because it's assumed the woman would refuse.” (Mi3).

“We don't force her unless it's a matter of life or death, we don't force her” (Mi2).

“We are a very heterogeneous group of gynaecologists, each with our own approach to practice. That's simply the way it is” (O2).

One professional stated that measures are taken to protect themselves from possible accusations of obstetric violence.

“For example, written consent is obtained for cesarean sections; however, at present, there is no written consent for instrumental deliveries. Discussions on this matter have begun, primarily from a defensive standpoint, in the context of obstetric violence. Currently, informed consent is obtained for caesarean sections and epidurals at the time of birth, but no other informed consent is provided, and patients sign these documents.” (O4).

##### 3.2.1.4 The difficulty of multidisciplinary work

A midwife recounts the difficulty of working with an anaesthesiologist and mentions that women often suffer pain due to delays in administering the epidural.

“If the resident is in the operating room, the other (senior) anaesthesiologists don't wake up. Therefore, we have the woman screaming for two, three, four hours. Sometimes they say (anaesthesiologists): ‘Yes, I'll come up later’. (Women) They go through a terrible experience, and this happens to us quite often. For me, this is obstetric violence” (Mi1).

Some midwives are forced or coerced into performing acts they consider unnecessary by obstetricians, which creates discomfort for them.

“There was one instance when I was forced to perform an episiotomy, even though I believed it wasn't necessary. It made me feel awful.” (Mi2).

“There was a time when I was asked to perform a Kristeller's manoeuver, and I refused and left the birth room. I thought to myself, “You're the one who might end up in court."” (Mi3).

A mother reports an instance of mistreatment between the anaesthesiologist and the midwife:

“The anaesthesiologist treated both me and the midwife very poorly.. He attempted to insert the needle three times and blamed us both: me for not positioning myself as he wanted, and the poor midwife for not holding me properly” (M4).

#### 3.2.2 The mothers' point of view

**Figure 3:**
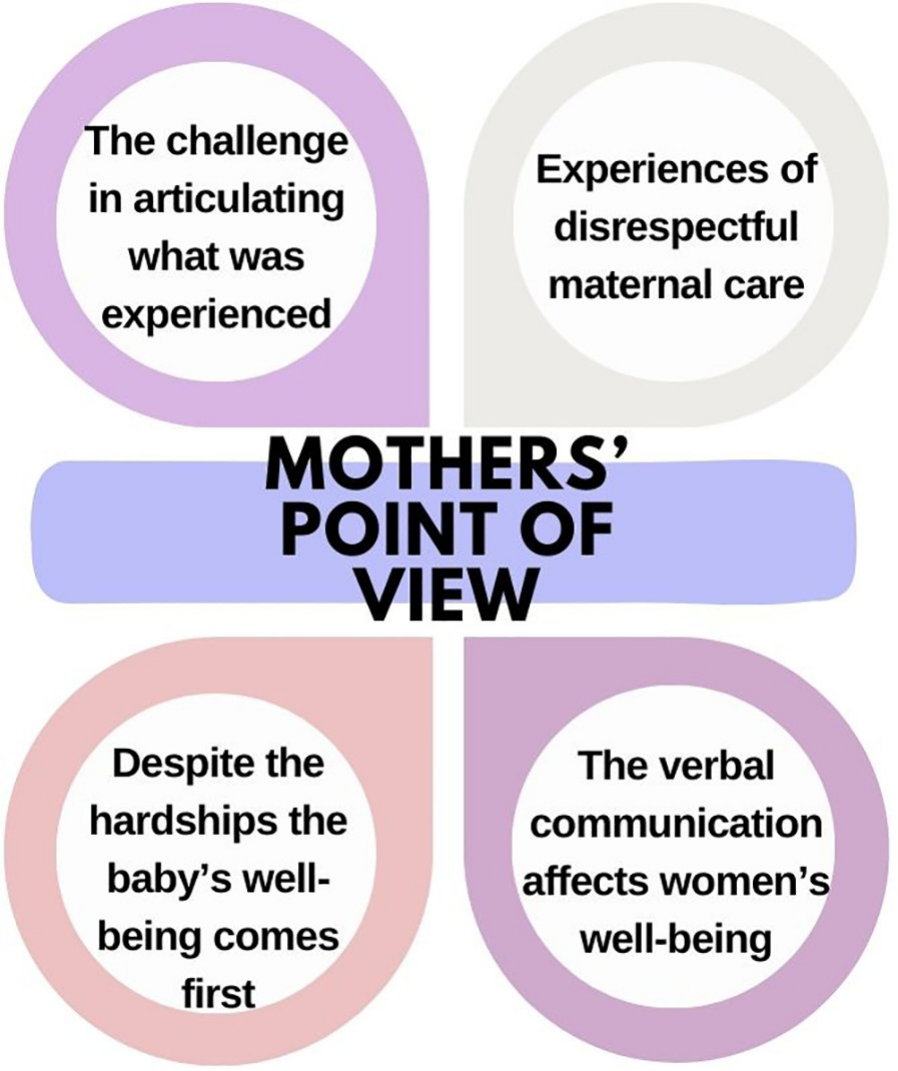
Themes representing the mothers’ point of view of obstetric violence.

Themes representing the mothers' point of view of obstetric violence are shown in Figure 3.

##### 3.2.2.1 The challenge in articulating what was experienced

When directly asked if they believe they were violated during their birthing process, some women report that the term is too strong and deny it, even though what they subsequently describe aligns with experiences of disrespectful maternal care.

“No, I don't want to go to this extreme because I didn't feel that way. I felt that they disrespected me, that they violated my privacy, that they didn't take me into account. I felt ignored. I felt bad, that they weren't respecting me” (M1).

“Respectful maternal care? No, I carry trauma from it. I wouldn't have another child. Pregnancy, childbirth, and breastfeeding, all together, for me was a traumatic experience. I would change the entire birth team. Violated might sound like a strong word, but I felt like they wanted to silence me. It seemed like she (the midwife) wasn't willing to have much patience with me, and I felt like insulted” (M3).

##### 3.2.2.2 Experiences of disrespectful maternal care

A woman recounts feeling pressured into receiving an epidural:

“They did ask me about the epidural, and I told them I wasn't sure. When she noticed I was complaining a bit about pain, she said ‘no, no, it's better if we give it to you because you won't be able to handle it’. I think I might have been able to manage without it, but she insisted so much. It was like she was saying (imitating) ‘Mommy, we'll just give it to you. Trust me, listen to me” (M5).

Some non-native women reported receiving poor care and believe it may have been due to their skin colour:

“And they kept spreading my legs, kept doing vaginal examinations, kept leaning on me, and started talking among themselves. I feel that if I had been from here or had lighter skin, they might not have behaved that way” (M1).

Several women agree that the care provided by the anaesthesiologist was poor, and part of their negative memories is related to the anaesthesia administration.

“The worst moment was the anaesthesiologist who treated you terribly, terribly, terribly, horribly” (M4).

“The anaesthesiologist's moment was a drama. If the epidural's lady had treated me differently, I wouldn't have been in such nervous and distressed. That woman was incredibly cold and unfeeling” (M8).

Mothers, like professionals, also report that at times, an excessive number of healthcare professionals were present during childbirth.

“I am very satisfied. I also have to say that I don't know if there were 7 professionals present during the birth “ (M6).

##### 3.2.2.3 The verbal communication affects women’s well-being

A woman was criticized by some professionals for the number of children she had and was advised not to have any more.

“The third one already? Let this be the last one, huh?” Look, it's my life, isn't it? You're not coming to support them. Keep those comments to yourself. And then they look at my age: 29? So young! 3? That's enough, huh? No more, huh?” (M1).

Some mothers report being forbidden to scream, while others felt ignored as professionals discussed their concerns during a caesarean section.

“They did tell me, don't scream. That's how they said it, don't scream” (M3).

“The girls who were putting in the staples, they were talking about their weekend or something about their boyfriend. And I'm not interested, I'm in a critical moment, this is an operation. The care here wasn't as professional as it should be. I experienced it very negatively during the birth, the way of treating me roughly, the vaginal examinations, the movements” (M1).

Some mothers stated that their sense of respect varied depending on which professionals attended to them.

“I noticed a big difference depending on which professional attended me” (M6).

Some women were unaware of certain procedures performed on their newborns, while others would have appreciated being asked for consent beforehand.

“(Administration of vitamin K) I didn't realize they had given that to him. Do they give it when you give birth? I don't know, they don't ask you” (M5).

“They performed a technique to position the newborn, which is good, right? But I would have liked to be asked first, to be informed..” (M1).

##### 3.2.2.4 Despite the hardships, the baby's well-being comes first

Finally, despite everything the mothers tell us, the most important thing for them is the well-being of their baby.

“The care before labour and postpartum was great. The birth was terrible. The important thing is the baby's well-being” (M3).

“For me, the most important thing is the girl, I mean, if she's okay, then perfect” (M7).

## 4 Discussion

This study explores the experiences and perceptions of maternal care, as well as the phenomenon of obstetric violence.

**Figure 4:**
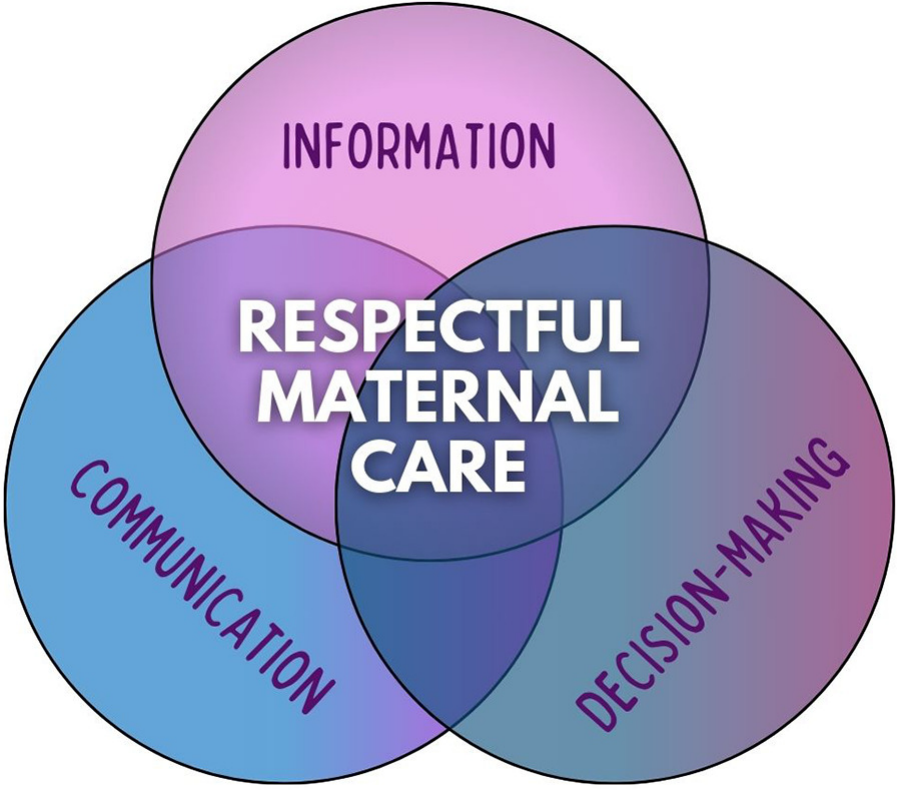
Categories that interact to describe respectful maternal care.

The results obtained from the analysis of the discourses have allowed us to identify and relate categories that interact to describe respectful maternal care (Figure 4). When women receive accurate and comprehensive information about the entire maternal process, combined with effective communication that fosters a strong therapeutic relationship, they are better equipped to make informed decisions, feel in control of the situation, and, consequently, perceive that they have received respectful maternal care.

Regarding information, healthcare is undergoing a shift in the patient model. In obstetrics, pregnant women are becoming increasingly empowered and informed, while also growing more critical and cautious toward the healthcare system. Our results align with those obtained by Jovell (32) and Fernández-Aranda (33).

In our study, both women and professionals agree that there is a lack of information during healthcare assistance in pregnancy, childbirth, and postpartum according to authors such as Marrero and Brüggemann (11) and Khalil et al. (20) As a result, mothers often seek information from unreliable sources to meet their immediate needs as supported by Sayakhot et al. (34). This information, typically found on the internet, plays a crucial role in decision-making and can raise expectations that, if unmet, may lead to frustration and dissatisfaction. Lagan et al. (35) found same results.

The main reason for the lack of information is the shortage of time during clinics, which both professionals and mothers report. They state that they are attended to in a rush and cannot resolve all their doubts during the clinic. Our results are consistent with those reported by Nagesh et al. (12).

Concerning communication, the mothers in our study are not satisfied with the communication they receive from obstetrics and anaesthesia professionals (19, 36). The WHO recommends effective communication in birth care (1). Good communication reduces anxiety, improves self-care, and increases adherence to treatments (37). Professionals state that without good communication, it is not possible to establish a therapeutic relationship, and this can be affected by the healthcare professional's communication skills. Education on empathetic and respectful communication should be included at university because are not innate skills (38). The patient must perceive the professional as an honest, trustworthy, warm, and attentive person who validates them unconditionally and accepts them to establish a therapeutic relationship (39). Additionally, it allows mothers and professionals to address problems that may arise during labour and birth (40).

Some of the participant mothers in our study report that they did not feel well-treated due to incidents such as the professionals entering in a clinic without introducing themselves, discussing personal matters during critical moments, or making comments about the number of children. In the public healthcare system, particularly in obstetrics, it is difficult to establish this therapeutic relationship due to the absence of a reference professional. The language and cultural barriers also affect communication and quality of care (41) and they hinder the ability to properly convey information and enable the mother to make informed decisions (13).

Related to decision-making, a birth plan is a document that outlines the preferences of pregnant women, facilitates communication with professionals, promotes decision-making, and improves satisfaction (42). Although it is associated with fewer interventions and better health outcomes for both the mother and baby, not all women create one, and it is often disregarded (43). Some professionals believe that the birth plan can raise expectations or idealize childbirth, particularly regarding natural births, which reflects the normalization of medicalization in obstetrics.

In obstetrics, interventions such as episiotomies are often carried out without the prior consent of mothers (11, 20). Both American College of Obstetricians and Gynaecologists (ACOG) and current Spanish regulations require that the clinical indications for interventions be communicated, and that the woman consents to the procedure through written informed consent (44, 45). Mothers have the right to refuse treatment or intervention, even if the professional considers it to be in their best interest (46). It is essential to foster shared clinical decision-making, avoid abuses of power by institutions and professionals, and preserve women's autonomy (47). Increasingly, mothers are rejecting paternalistic relationships and seeking a more horizontal one, based on mutual collaboration (32, 33).

When exploring the term “Obstetric Violence” (OV) and its definition, the obstetricians in our study consider the term to be offensive because it implies intentionally causing pain. Some mothers feel that the term “feeling violated” is too harsh, but they have experienced being ignored, disrespected, having their privacy violated, and being treated with disregard. Other studies use terms such as “*mistreatment during childbirth*” or “*disrespect and abuse during childbirth*” (4, 14, 19, 21).

Regarding the definition, obstetricians do not provide a specific one but believe that, in general, people do not distinguish between actions that constitute obstetric violence and those that are considered medical indications. Midwives, however, view OV as acts, techniques, or comments that affect the mother's sensitivity or are unnecessary. It is a concept that holds many interpretations and can be seen as such when techniques are practiced that the mother does not wish to undergo. According to the literature, obstetric violence is defined as the abuse or mistreatment by a healthcare provider of a female engaged in fertility treatment, preconception care, pregnancy, childbirth, or postpartum; or the performance of any invasive or surgical procedure during the full span of the childbearing continuum without informed consent, coerced, or in violation of refusal (22). Some professionals do not consider performing medical acts without consent to be obstetric violence, as they do so to ensure the safety of the woman and the baby (11). It also encompasses unnecessary but routine medical interventions, such as episiotomies or instrumental births for teaching purposes or to accelerate birth (21).

Our findings align with the feminist conceptualization of obstetric violence as a form of structural and epistemic violence against women's bodies and autonomy (15, 48, 49). Feminist scholars have argued that obstetric violence is not merely the result of individual malpractice but is embedded within broader patriarchal structures that shape obstetric care. For instance, Shabot and Korem (48) emphasizes the phenomenological experience of violation during childbirth, while Pickles (15) highlights the legal and ethical challenges in addressing obstetric violence as a human rights issue. Incorporating these perspectives allows for a deeper understanding of the power dynamics, dehumanization, and systemic inequalities that underpin the participants' experiences reported in this study.

In relation to professionals' experiences, obstetricians and midwives state that OV occurred more frequently in the past but is becoming less common in birth rooms. They agree that many procedures need to be changed because some acts are performed routinely, and the woman's decision carries little weight. As a result, these routine procedures can trivialize actions such as caesarean sections or instrumental deliveries (12). Obstetricians acknowledge that some practices are based on low-quality scientific evidence (50). The main issue is that most obstetricians and midwives have been trained in a system where OV was normalized, making it more difficult for them to detect and reflect upon it (51)*.* The ACOG suggests practicing techniques such as operative vaginal delivery, postpartum haemorrhage management, shoulder dystocia management, perineal laceration repair, conventional laparoscopic procedures, and robotic surgery through simulation rather than in real clinical scenarios (52)*.*

The narratives reveal that, unlike midwives, obstetricians do not identify situations that could be considered obstetric violence (OV). This difference may stem from obstetricians still rejecting the term. Midwives report experiences where pain management has been ineffective, either because anaesthesia was not administered or was given too late (16, 17). This can generate feelings of frustration, as many women associate the quality of care, they received with how pain was relieved (12). On the other hand, some mothers reported feeling coerced into receiving epidural analgesia (53), despite the WHO recommending that maternal preferences be assessed (1).

Both midwives and mothers report an excess of staff during deliveries, as well as unprofessional behaviour, such as engaging in personal conversations. While the literature does not specify the exact number of professionals that should be present during childbirth, the WHO recommends having a sufficient and competent team (1).

Some midwives admit to performing the Kristeller's manoeuver because they considered it necessary, despite the WHO recommending against this technique (1). Kristeller's has been associated with fractures, brain damage, brachial plexus injuries in newborns, as well as 3rd-4th degree vaginal lacerations, rib fractures, uterine rupture, placental abruption, and postpartum haemorrhage in mothers (54)*.*

Regarding non-consensual practices, some professionals exercise their autonomy without considering the woman's will (55). To avoid future lawsuits for obstetric violence, healthcare centers create unnecessary and multiple informed consents as a form of defensive medicine (56). In our study, professionals reported creating a protocol requiring women to sign a form if they refuse the routine insertion of a peripheral intravenous line. When mothers describe mistreatment, they often refer to communication and verbal language rather than specific medical practices. This includes discrimination based on skin colour, comments about fertility, and the restriction of expressing pain freely through screaming (12, 13, 19, 20, 36). The attitudes with which professionals treat women are crucial to mothers' perceptions of the care received (12). The WHO suggests that professionals should treat women kindly for a positive birth experience (1). However, mothers are often treated in a hostile, unempathetic, unprofessional, and authoritarian manner (16, 18). Midwives report occasionally feeling obligated to perform interventions they consider unnecessary. One study noted a midwife stating, “*Sometimes you feel you have to protect women from insensitive healthcare professionals*,” referring to obstetricians (8). Another figure mentioned by both mothers and midwives is the anaesthesiologist, due to the verbal mistreatment they sometimes exhibit.

Finally, it is important to highlight that the maternal care received impacts women's autonomy and integrity over their bodies and sexuality (57)*.* The prioritization of optimizing personnel and resources, as well as standardizing childbirth care, could increase the likelihood of experiencing OV (13). Women who have not received respectful maternal care and have experienced OV may exhibit symptoms of anxiety, panic attacks, postpartum depression, suicidal thoughts, marital breakdown, sexual dysfunction, incontinence, emotional disconnection from the baby, among others (2).

Nevertheless, despite having experienced what is considered a traumatic birth, both women and professionals often focus on the well-being of the newborn. A birth is typically regarded as successful if the baby is born healthy, with the mother's well-being often relegated to a secondary concern.

## 5 Strengths and limitations

This study is the first to compare the narratives of both women and healthcare professionals, ensuring data saturation from two independent sources. Additionally, it features a highly diverse sample of participants. Among the women, two are non-native, and they vary in educational background, parity, and childbirth experiences. Similarly, the healthcare professionals bring diverse perspectives, differing in years of experience, workplace settings, and gender, thus enriching the study with a broad and comprehensive viewpoint.

The most significant limitation of our study is the inability to interview women who face language barriers, despite them representing 30% of those attending our birth rooms. Moreover, we recognize that linguistic and cultural barriers are key factors that may contribute to obstetric violence. Future studies should include mediators to eliminate this exclusion criterion. Another limitation is that our findings are based on data from a single region in Catalonia, meaning the experiences of women in other regions may differ.

## 6 Conclusion

The care provided to women during pregnancy, childbirth, and postpartum is undergoing significant changes that highlight the limitations of the traditional obstetric model. The growing demand for more conscious, personalized, and respectful care from women calls for a review and update of current models and protocols to promote evidence-based, respectful assistance, moving away from the pathologization of physiological processes. An obstetric model that guarantees women's autonomy and moves away from defensive medicine not only improves their experience but also contributes to safer and more efficient care.

The difficulty in implementing changes in obstetrics lies, in part, in the controversy and debate surrounding the concept of obstetric violence. The term “violence” is uncomfortable for both mothers and professionals, as it evokes the intention to cause harm. To move towards a model of respectful maternal care, it is crucial to overcome terminological disputes and implement public policies that ensure a more respectful care model. It is essential to incorporate debate and reflection on gender issues in students, rethinking childbirth care from a humanistic approach that focuses on women. We must move away from the old obstetric model and ensure respectful maternal care, not only through public policies but also through training programs that integrate this sensitivity. In the same vein, it is important to ensure a positive birth experience for women, avoiding the subordination of maternal health to fetal well-being.

Achieving this goal is a collective responsibility that must be assumed by institutions and professionals to ensure universal, respectful obstetric care.

## Data Availability

The original contributions presented in the study are included in the article/Supplementary Material, further inquiries can be directed to the corresponding authors.
